# GSTZ1‐1 downregulates Wnt/β‐catenin signalling in hepatocellular carcinoma cells

**DOI:** 10.1002/2211-5463.12769

**Published:** 2019-12-10

**Authors:** Chong Lei, Qiujie Wang, Ni Tang, Kai Wang

**Affiliations:** ^1^ Key Laboratory of Molecular Biology for Infectious Diseases (Ministry of Education) Department of Infectious Diseases Institute for Viral Hepatitis The Second Affiliated Hospital Chongqing Medical University China

**Keywords:** GSTZ1‐1, HCC, hepatocellular carcinoma, RNA‐Seq, Wnt, β‐catenin signalling pathway

## Abstract

Glutathione S‐transferase Zeta 1‐1 (GSTZ1‐1), an enzyme involved in the catabolism of phenylalanine and the detoxification of xenobiotics, plays a tumour suppressor role in hepatocellular carcinoma (HCC), but the underlying mechanism remains largely unknown. Here, we further explored the function of GSTZ1‐1 in HCC through transcriptome analysis by RNA sequencing. The analysis revealed that 223 genes were upregulated and 290 genes were downregulated in GSTZ1‐1‐overexpressing Huh7 cells. Gene Ontology analysis showed that these differentially expressed genes (DEGs) were highly enriched for protein phosphorylation, cell cycle arrest and metabolic processes. Pathway analysis revealed that metabolic pathways were the predominant enriched pathways among the upregulated genes, while the TGF‐β and Wnt/β‐catenin signalling pathways were prominent in the downregulated clusters. Pathway interaction networks also showed that the Wnt/β‐catenin pathway was located in the centre of the cluster. The expression levels of selected DEGs were validated by qRT‐PCR, and Wnt/β‐catenin involvement was validated by luciferase assays, western blotting and immunohistochemical analysis *in vitro* and *in vivo.* These results provide a comprehensive overview of the transcriptome in GSTZ1‐1‐overexpressing Huh7 cells and indicate that GSTZ1‐1 may play a tumour suppressor role by inactivating the Wnt/β‐catenin signalling pathway.

## Graphical abstract

Glutathione S‐transferase Zeta 1‐1 (GSTZ1‐1), an enzyme involved in the catabolism of phenylalanine and the detoxification of xenobiotics, plays a tumour suppressor role in hepatocellular carcinoma. Here, we found that GSTZ1‐1 could downregulate Wnt/β‐catenin signalling in hepatoma cells.

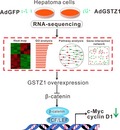

**Abbreviations**
DEG: differentially expressed genes
GO: Gene Ontology
HCC: hepatocellular carcinoma
IHC: immunohistochemical
KEGG: Kyoto Encyclopedia of Genes and Genomes
NASH: nonalcoholic steatohepatitis
RNA‐seq: RNA sequencing

## Introduction

Liver cancer is the sixth most common human malignancy and ranked fourth in global mortality in 2018 1. Hepatitis B virus or hepatitis C virus infection, nonalcoholic steatohepatitis (NASH) and alcohol consumption are the main risk factors for liver cancer 2. Recently, the roles of metabolic disorders in tumorigenesis have been increasingly researched. Many studies have reported that metabolic disorders such as obesity, diabetes and NASH can trigger hepatocellular carcinoma (HCC) 3, 4, 5, 6.

Glutathione S‐transferase Zeta 1 was identified by sequence alignment and phylogenetic analysis approximately 20 years ago 7. GSTZ1‐1 is a 24‐kDa cytoplasmic protein that isomerizes maleylacetoacetate to produce fumarylacetoacetate, which is the penultimate step in phenylalanine (Phe) degradation; furthermore, GSTZ1‐1 can detoxify xenobiotics 8, 9, 10.

Importantly, our previous work and other data have shown that GSTZ1‐1 is downregulated in (HCC) and that it plays a tumour suppressor role in HCC progression by inhibiting the NRF2/IGF1R axis 11, 12. However, a full and comprehensive understanding of the molecular mechanism of GSTZ1‐1 in HCC development remains largely unknown.

High‐throughput RNA sequencing (RNA‐Seq) is a sequencing‐based tool that surveys the entire transcriptome at the cellular level 13. In the present work, we aimed to comprehensively identify differentially expressed gene (DEG) profiles related to GSTZ1‐1 overexpression in hepatoma cells. Huh7 cells, which exhibit relatively low endogenous GSTZ1‐1 levels, were infected with a recombinant adenovirus prior to RNA‐Seq analysis and DEG profiling. Gene Ontology (GO), pathway, pathway interaction network, gene interaction network and co‐expression network analyses were conducted to determine the potential associations between the identified DEGs and HCC. Our findings may help illuminate the molecular mechanisms underlying the tumour suppressor role of GSTZ1‐1 during HCC development.

## Materials and methods

### Antibodies, cell lines and plasmids

The following antibodies were used in this study: GSTZ1‐1 (Cat#GTX106109; GeneTex, Irvine, CA, USA), β‐catenin (Cat#RLM3403; Ruiying, Suzou, Jiangsu, China), c‐Myc (Cat#BS2462; Bioworld, Louis Park, MN, USA), cyclin D1 (Cat#2978; CST, Beverly, MA, USA), RB (Cat#BS1310; Bioworld) and β‐actin (Cat#BL005B; Biosharp, Hefei, Anhui, China). Huh7 cells were obtained from the Cell Bank of the Chinese Academy of Sciences (CBCAS, Shanghai, China). HepG2, SNU449 and HEK293T cells were obtained from the American Type Culture Collection (ATCC, Rockville, MD, USA). The cells were cultured in Dulbecco’s modified Eagle’s medium (HyClone, Logan, UT, USA) supplemented with 10% FBS (Gibco, Grand Island, NY, USA) and 100 units per mL penicillin and streptomycin under standard conditions (37 °C in a humidified 5% CO_2_ atmosphere).

### Adenovirus‐mediated GSTZ1‐1 overexpression

The full‐length cDNA of GSTZ1‐1 (NM_145870.2) was cloned into the shuttle vector pAdTrack‐TO4. The recombinant adenovirus AdGSTZ1‐1 was prepared as described previously 14. Huh7 cells were infected with AdGSTZ1‐1 to establish a GSTZ1‐1‐overexpression (GSTZ1‐1‐OE) cell model.

### CRISPR‐Cas9‐mediated knockout of GSTZ1‐1

A single guide RNA (sgRNA) targeting GSTZ1‐1 was designed, synthesized and then cloned into a lentiCRISPR‐v2 vector. HEK293T cells were treated with GSTZ1‐1 sgRNA or lentiCRISPR‐v2, pMD2.G and psPAX2 for lentiviral preparation. Then, HepG2 and SNU449 cells were infected with the collected lentiviral supernatants for 48 h. The transduced cells were selected with 1 μg·mL^−1^ puromycin and diluted to single‐cell suspensions in a 96‐well plate. Single clones were generated from T‐A clones for genotyping and confirmed by western blotting. The associated primer sequences are shown in Table 1.

**Table 1:** Sequences of the primers.

FZD4	Forward: TCCCACCACAGAACGACCA
	Reverse: AAGCCAGCATCATAGCCACA
FZD5	Forward: CGTGGGCAACCAGAACCT
	Reverse: GACCGTGTAGAGCAGCGTGA
FZD6	Forward: TCTGCTGTCTTCTGGGTTGG
	Reverse: GCTGTAGCTCCTGTGCTGGTT
WNT11	Forward: ACAACAGTGAAGTGGGGAGACA
	Reverse: ACCAGGTGCTTGCGGGT
VANGL2	Forward: CACGCATCGCCAAGGAC
	Reverse: GGGCACGCAGCACAAAG
NFAT5	Forward: GTGTTTGTGGGCAACGACTC
	Reverse: TGGAACCAGCAATTCCTATTCT
UGT2B11	Forward: GCAAACCTGCCAAACCCC
	Reverse: TATTCCCGTCAAATCTCCACA
EPHX1	Forward: CACCGCCAGGATCTTTTACA
	Reverse: GCCAAGAAACCTCCCGAAA
E2F2	Forward: CGTGCTGTTGGCAACTTTAA
	Reverse: GGCAGAGGGTGGAGGTAGAG
RB1	Forward: CAAGTTTCCTAGTTCACCCTTACG
	Reverse: CGGTCGCTGTTACATACCATCT
TFDP2	Forward: ATCAGAAGAACATTAGGCGAAGA
	Reverse: AAAGCGATTTGCTGTAGGAGAA
GSTZ1‐sg	Forward: CACCGCCCAGAACGCCATCACTTG
	Reverse: AAACCAAGTGATGGCGTTCTGGGC
GSTZ1‐seq	Forward: GGACCATGCAAGGGAGAA
	Reverse: TTAAGACGGTTTAGTGGGAGTG

### Top‐luc reporter assays

Huh7 cells infected with a GFP‐expressing adenovirus (AdGFP) and AdGSTZ1‐1 were transiently co‐transfected with a Top‐luc reporter plasmid and a pRL‐TK plasmid using Lipofectamine™ 3000 (L3000015; Invitrogen, Carlsbad, CA, USA). Firefly and Renilla luciferase activity was measured using a Dual‐Glo Luciferase Assay System (Promega, Madison, WI, USA). The relative firefly luciferase activity was normalized to the corresponding Renilla luciferase activity.

### RNA preparation and quantitative reverse‐transcription (RT)‐PCR (qRT‐PCR)

Total cellular RNA was extracted from cultured cells with TRIzol™ reagent (Invitrogen) and then reverse transcribed to generate cDNA using a PrimeScript™ RT Reagent Kit (Takara, Dalian, Liaoning, China) with random hexamers following the manufacturer’s procedures. qRT‐PCR was performed using iTaq™ Universal SYBR® Green Supermix according to the manufacturer’s manual. The primer sequences for qRT‐PCR are shown in Table 1.

### Western blotting

Total cellular protein was extracted with cell lysis buffer (Beyotime, Nantong, China) and then quantified by BCA assay. The protein lysates were separated by SDS/PAGE, transferred to polyvinylidene difluoride membranes, probed with the indicated primary antibodies and HRP‐conjugated secondary antibodies, and then detected by enhanced chemiluminescence.

### Patient tissues

Paired human HCC and nontumour tissues (NT) were obtained from eight patients who underwent surgery at the Second Affiliated Hospital of Chongqing Medical University between 2017 and 2018 approved by the Institutional Review Board of Chongqing Medical University. Informed written consent was obtained from all patients. The study methodologies conformed to the standards set by the Declaration of Helsinki.

### Immunohistochemical (IHC) analysis

Human HCC and paired NT sections were deparaffinized, and then, antigens were retrieved in 10 mm citric acid buffer (pH 6.0) by microwave. Subsequently, the section samples were penetrated with 0.5% Triton X‐100, and endogenous peroxidase was blocked with 3% hydrogen peroxide (Cat#ZLI‐9311; zsbio, Beijing, China). The sections were incubated overnight at 4 °C with anti‐β‐catenin (1 : 200) and anti‐GSTZ1(1 : 200) primary antibodies. The slides were processed with a DAB Substrate Kit (Cat#ab64238; Abcam, Cambridge, MA, USA) according to the manufacturer’s protocol and counterstained with haematoxylin.

### RNA‐Seq analysis

Total RNA was extracted from Huh7 cells infected with AdGFP or AdGSTZ1‐1 (*n* = 3 for each). RNA quality was assessed by a Nano Drop 2000 Bioanalyzer, and RNA‐Seq was performed by Shanghai Novel‐bio Company. The gene expression data have been deposited in the Gene Expression Omnibus (GEO) database under accession number http://www.ncbi.nlm.nih.gov/geo/query/acc.cgi?acc=GSE117822 15.

### DEG analysis

Differentially expressed genes were screened using the algorithm DESeq under a threshold of |log_2_(fold change)|≥ 0.585 with a false discovery rate (FDR) ≤ 0.05.

### GO analysis

Fisher’s exact test was used to classify the GO category (*P *≤ 0.01), and the FDR values were calculated to correct the *P*‐values.

### Pathway analysis

Pathway analysis was used to find significantly enriched pathways according to the Kyoto Encyclopedia of Genes and Genomes (KEGG) database. The upregulated and downregulated pathway categories with FDR values ≤0.05 are shown.

### Pathway interaction network and gene interaction network analyses

The KEGG database was used to build a network of upregulated and downregulated pathways and genes according to their relationships with each other using a threshold of *P* ≤ 0.05.

### Co‐expression network analysis

Gene co‐expression networks were used to search for interactions among genes as described previously 16. For each pair of genes, Pearson’s correlation coefficient was calculated and significantly correlated pairs were chosen for construction of the network 17. In network analysis, the degree of centrality is the simplest and most important measure of the relative importance of a gene within a network. The degree of centrality is defined as the number of links one node has to another 18. The *k*‐core for each specific gene, indicating its hub status, was used to identify the core regulatory factors in networks. The degree difference (DifDegree) and *k*‐core difference (DifKcore) between two classes of samples were used to locate the core regulatory factors in this study. Genes with a DifDegree ≥ 12 and a DifKcore ≥ 8 were considered to be core regulatory factors 19, 20.

### Statistical analysis

Statistical data are shown as the means and standard deviations (SDs). *P*‐values were calculated by two‐tailed Student’s *t*‐tests using graph‐pad prism 6.0 software (GraphPad‐Prism Software Inc., San Diego, CA, USA), and a *P* ≤ 0.05 was defined as significant in this study.

## Results

### Analysis of DEGs identified with RNA‐Seq

**Figure 1:**
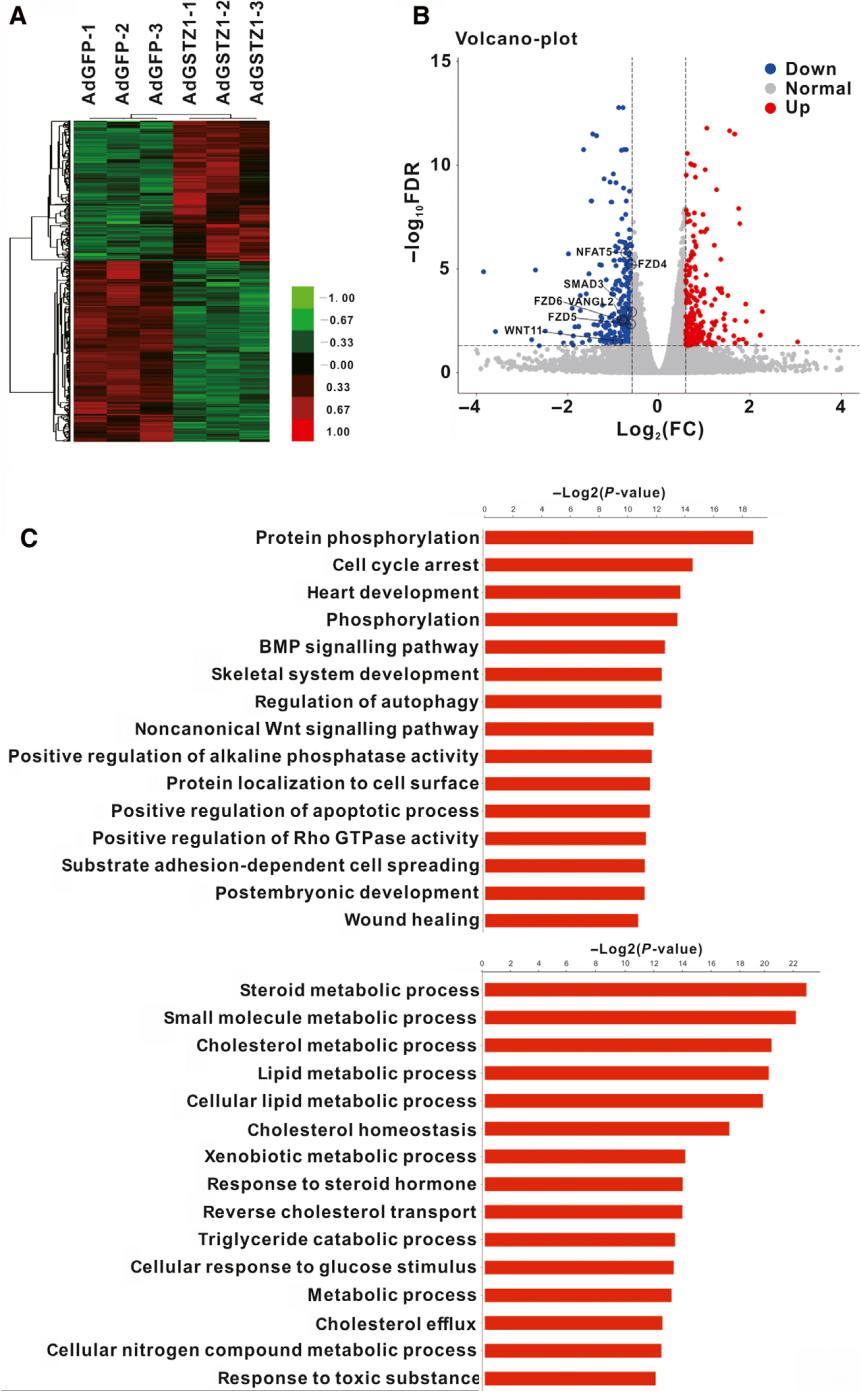
Identification of DEGs between Huh7 cells overexpressing GSTZ1‐1 and control Huh7 cells and GO analysis of the significant DEGs. (A) Clustering of DEGs showing transcript enrichment, which is encoded in the heat map from low (green) to high (red). (B) Volcano plot representing whole‐transcriptome changes in GSTZ1‐1‐overexpressing Huh7 cells (C) The top 15 downregulated (top) and upregulated (bottom) biological process (BP) terms are shown. *P* < 0.01 for all significant BP terms.

To further assess the function of GSTZ1‐1 in HCC, we performed RNA‐Seq to identify the DEGs between GSTZ1‐1‐overexpressing hepatoma cells and control cells. The DESeq algorithm was used to identify DEGs between the AdGSTZ1‐1 group and the AdGFP control group. The thresholds for DEG detection in this study were an FDR ≤ 0.05 and a│log_2_FC│≥ 0.585 (GSTZ1‐1‐OE vs control). A heat map (Fig. 1A) and a volcano plot (Fig. 1B) were constructed to display the comprehensive gene expression changes that were associated with GSTZ1‐1 overexpression. In total, 513 DEGs were identified following GSTZ1‐1 overexpression. Of these genes, 223 were upregulated and 290 were downregulated in GSTZ1‐1‐overexpressing Huh7 cells.

### GO analysis

To investigate the exact impacts of these DEGs on HCC development, GO analysis was used for gene annotation. A total of 304 GO terms were enriched among all downregulated DEGs. Among the upregulated DEGs, 292 GO terms were enriched. The enriched biological processes for the downregulated genes included protein phosphorylation and cell cycle arrest. However, the significant biological processes enriched for the upregulated genes were mainly metabolic processes, such as steroid metabolism, small molecule metabolism, xenobiotic metabolic process and responses to toxic substances (Fig. 1C).

### Pathway analysis and pathway interaction network analysis

**Figure 2:**
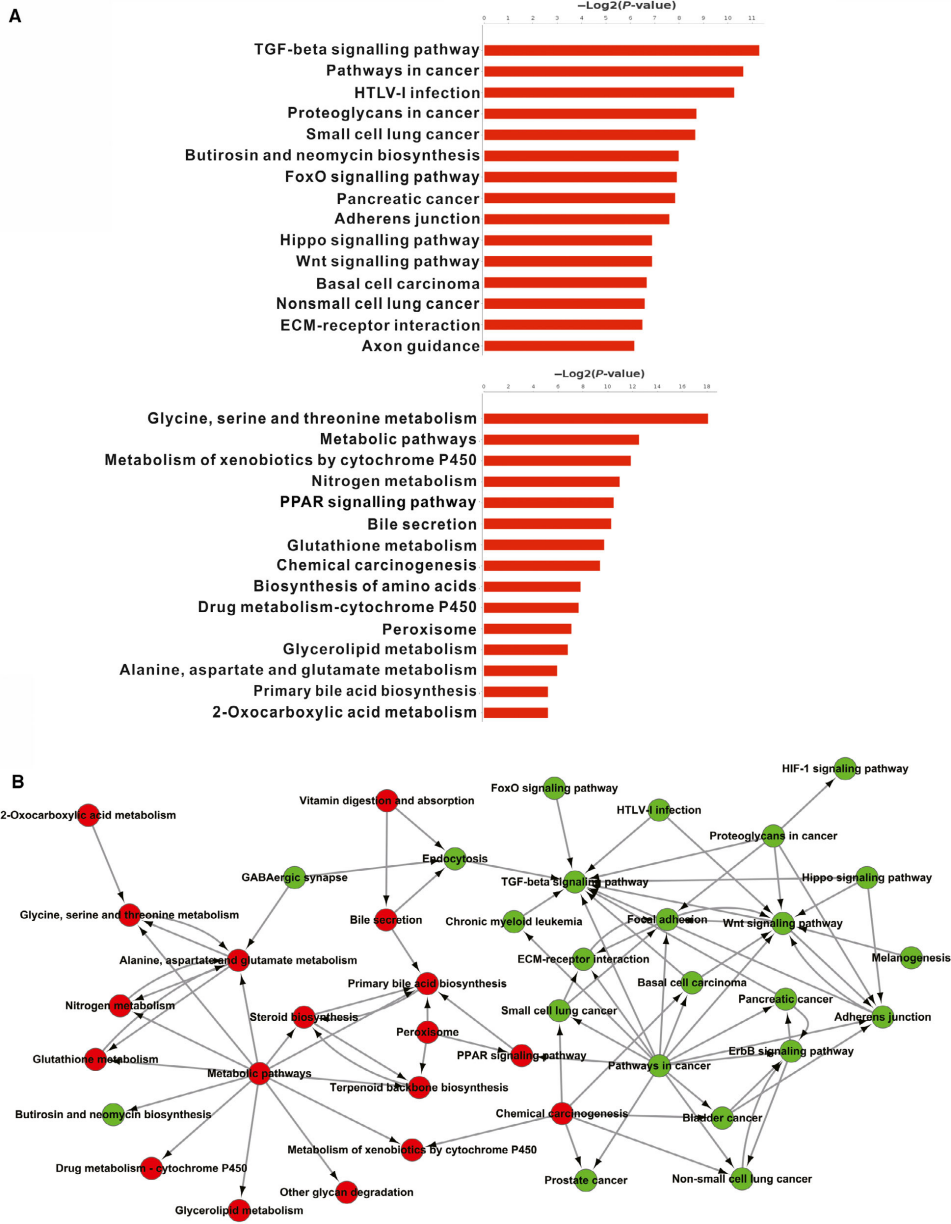
Pathway enrichment and pathway interaction network analysis. (A) The top 15 downregulated (top) and upregulated (bottom) pathways are shown. (B) Pathway interaction network. Red and green, represent upregulated and downregulated pathways, respectively.

The KEGG database was used to annotate the identified DEGs. Importantly, downregulated pathway terms, such as the TGF‐β signalling and Wnt/β‐catenin signalling pathway terms, are tightly associated with the development of liver cancer 21, 22. Among the upregulated pathways, the glycine, serine and threonine metabolism pathway was the most enriched, followed by the pathway for metabolism of xenobiotics by cytochrome P450 (Fig. 2A). A pathway interaction network was established for analysis of the associations of these pathways (Fig. 2B). As expected, we found that most upregulated metabolic pathways were located in the centre of the network, indicating metabolic pathway as the key upregulated pathway. Among the downregulated pathways, the Wnt and TGF‐β pathways were associated with many other pathways, suggesting that these were core downregulated pathways regulated by GSTZ1‐1 in HCC.

### DEG interaction network analysis

**Figure 3:**
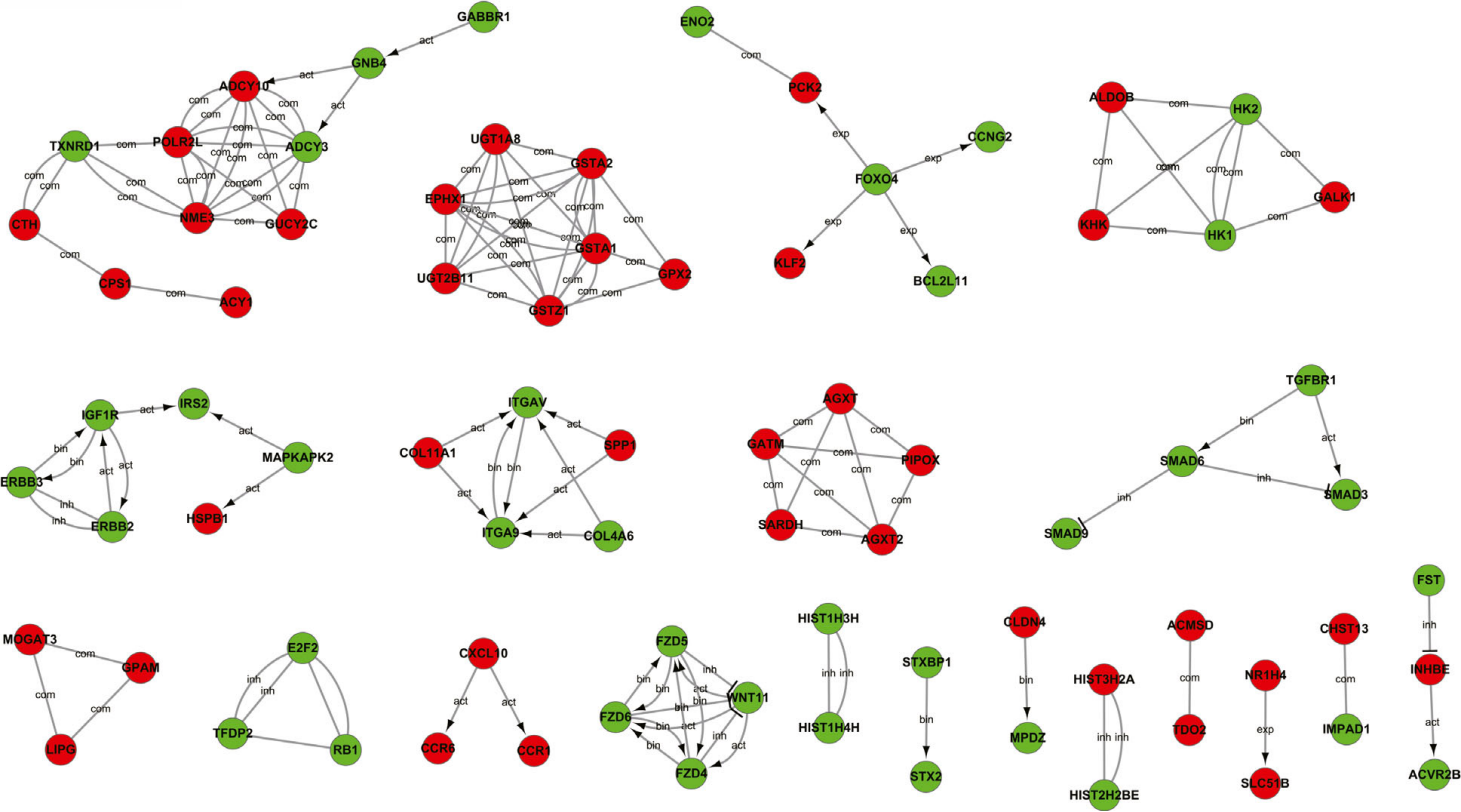
Gene interaction network analysis. The red and green circles represent upregulated and downregulated genes, respectively, in Huh7 cells overexpressing GSTZ1‐1. ‘Act’, activation; ‘inh’, inhibition; ‘bin’, binding; ‘com’, compound; ‘exp’, expression.

**Figure 4:**
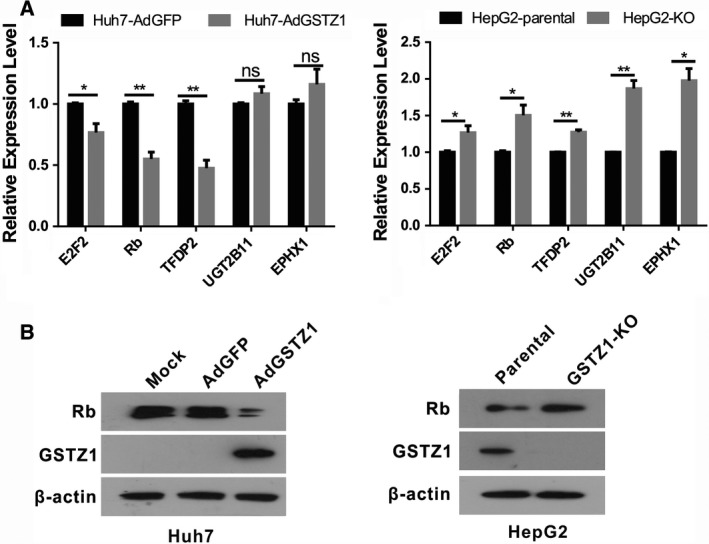
Validation of the gene interaction network analysis. (A) mRNA expression levels of cell proliferation‐related genes including E2F2, RB1 and TFDP2, metabolism of xenobiotic‐related genes including UGT2B11 and EPHX1 in GSTZ1‐1‐overexpressing and knockout cells. (B) Western blot analysis of endogenous Rb protein levels in GSTZ1‐1‐overexpressing and knockout cells. All error bars are the mean ± SD. All of the qRT‐PCR and western blot data are representative of three independent experiments. *P < 0.05, **P < 0.01, as determined by two‐tailed Student’s *t*‐test. ‘ns’ indicates ‘no significance’.

The DEGs identified in this study were used to establish a gene interaction network to determine the associations between the DEGs. The relationships between DEGs included activation/phosphorylation, binding/association, expression, inhibition, dissociation and compound relationships. The TGF‐β pathway‐related genes *TGFBR1, SMAD3, SMAD6, BMP8A, SMAD9, FST *and* ACVR2B* were downregulated. Similarly, we found that the Wnt/β‐catenin pathway‐related genes *FZD4, FZD5, FZD6 VANGL2, NFAT5* and *WNT11* were also downregulated. Notably, the cytochrome P450‐mediated xenobiotic metabolism‐related *UGT2B11, GSTA2, EPHX1, SULT2A1, UGT1A8 and GSTA1* were upregulated and they interacted with each other (Fig. 3) 20. To validate the reliability of the RNA‐Seq data, we observed the mRNA levels of *E2F2, Rb and TFDP2* in Huh7 GSTZ1‐1‐overexpressing and HepG2‐knockout cells by qRT‐PCR. We found that overexpression of GSTZ1‐1 reduced the mRNA expression of these genes, whereas knockout of GSTZ1‐1 had the opposite effect. We further detected Rb protein expression by western blot analysis and obtained the same results. As controls for GSTZ1‐1 function, *UGT2B11 and EPHX*1 (the metabolism of xenobiotics‐related genes) mRNA levels were detected by qRT‐PCR (Fig. 4A,B).

### Co‐expression network analysis

**Figure 5:**
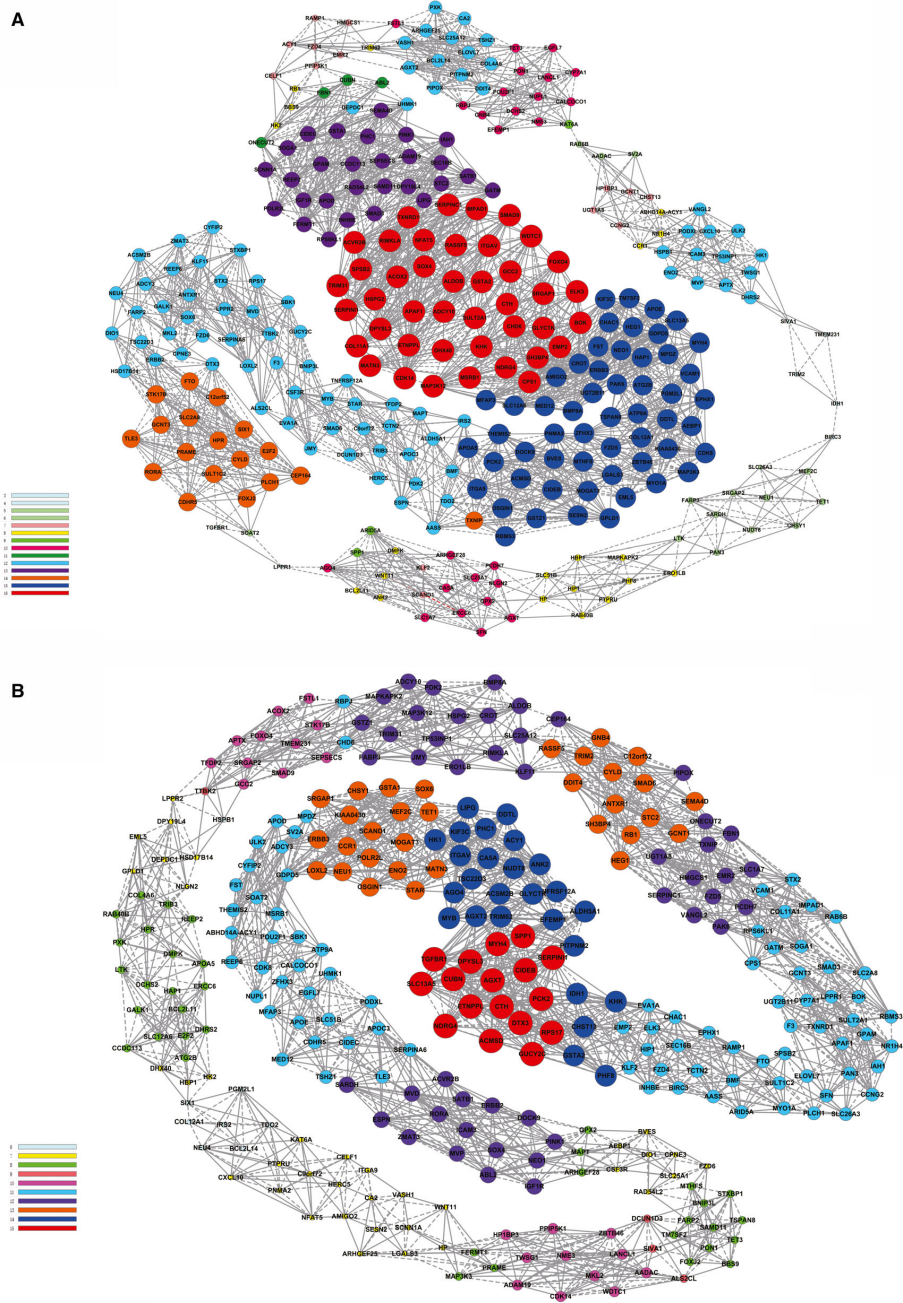
Gene co‐expression network. (A) Gene co‐expression networks for samples from control Huh7 cells. (B) Gene co‐expression networks for samples from GSTZ1‐1‐overexpressing Huh7 cells. A solid line indicates a positive correlation, and a dashed line indicates a negative correlation.

Next, a co‐expression gene network was constructed to analyse the complex relationships among the DEGs. The co‐expression gene networks of the GSTZ1‐1 group and the GFP control group differed significantly. The co‐expression network of the control group comprised 314 network nodes and 2698 connections, including 1145 that were negative connection and 1553 positive connection (Fig. 5A). Similarly, the network of the GSTZ1‐1 group contained 314 network nodes and 2500 connections, including 1399 that were positive, and 1101 that were negative (Fig. 5B). Differentially co‐expressed genes with DifDegree values ≥ 12 and DifKcore ≥ 8 values were defined as the core regulatory factors in the network. Based on the above criteria, *IDH1, TGFBR1, TRIM2, BIRC3* and *NUDT8* might play pivotal roles in the interactions 19, 20.

### Validation of the correlation between GSTZ1‐1 and the Wnt/β‐catenin pathway

**Figure 6:**
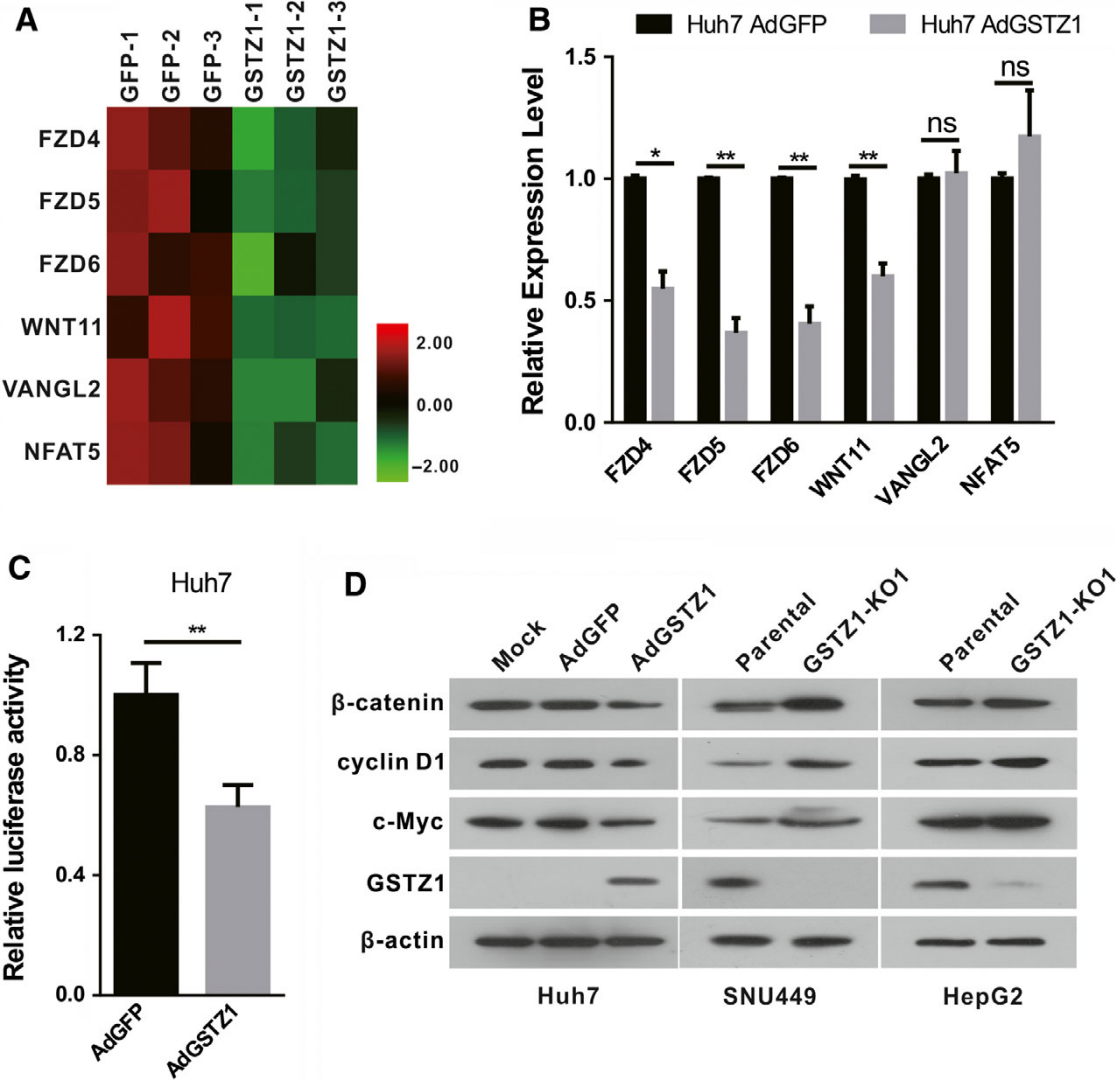
Validation of the Wnt/β‐catenin signalling pathway. (A) Heat map of downregulated genes involved in Wnt/β‐catenin signalling. (B) Six representative DEGs (*FZD4, FZD5, FZD6, WNT11, VANGL2 and NFAT5*) detected by RNA‐Seq were confirmed using qRT‐PCR. (C) GSTZ1‐1‐overexpressing Huh7 cells transfected with Top‐luc and Renilla pRL‐TK plasmids were subjected to dual luciferase assays 36 h after transfection. (D) Western blot analysis of the expression of β‐catenin, cyclin D1 and c‐Myc in GSTZ1‐1‐overexpressing Huh7 cells and HepG2, SNU449 knockout cells. All error bars are the mean ± SD. All of the qRT‐PCR and western blot data are representative of three independent experiments. *P < 0.05, **P < 0.01, as determined by two‐tailed Student’s *t*‐test. ‘ns’ indicates ‘no significance’.

According to the results described above, the Wnt/β‐catenin pathway was downregulated and was in the centre of the pathway interaction network. The six downregulated DEGs (*FZD4, FZD5, FZD6, WNT1, VANGL2 and NFAT5)* (Fig. 6A) involved in the pathway were analysed by qRT‐PCR (Fig. 6B), and the results were consistent with the RNA‐Seq results. Considering the pivotal role of Wnt/β‐catenin signalling in hepatocarcinogenesis, we further explored whether overexpression of GSTZ1‐1 suppressed Wnt/β‐catenin signalling. Indeed, GSTZ1‐1 overexpression significantly reduced the activity of β‐catenin as determined by the Top‐luc reporter assay (Fig. 6C). Furthermore, GSTZ1‐1 overexpression decreased the protein expression levels of β‐catenin, as well as those of the downstream targets c‐Myc and cyclin D1 in Huh7 cells, whereas knockout of GSTZ1‐1 increased β‐catenin, c‐Myc and cyclin D1 protein levels in HepG2 and SNU449 cells (Fig. 6D).

**Figure 7:**
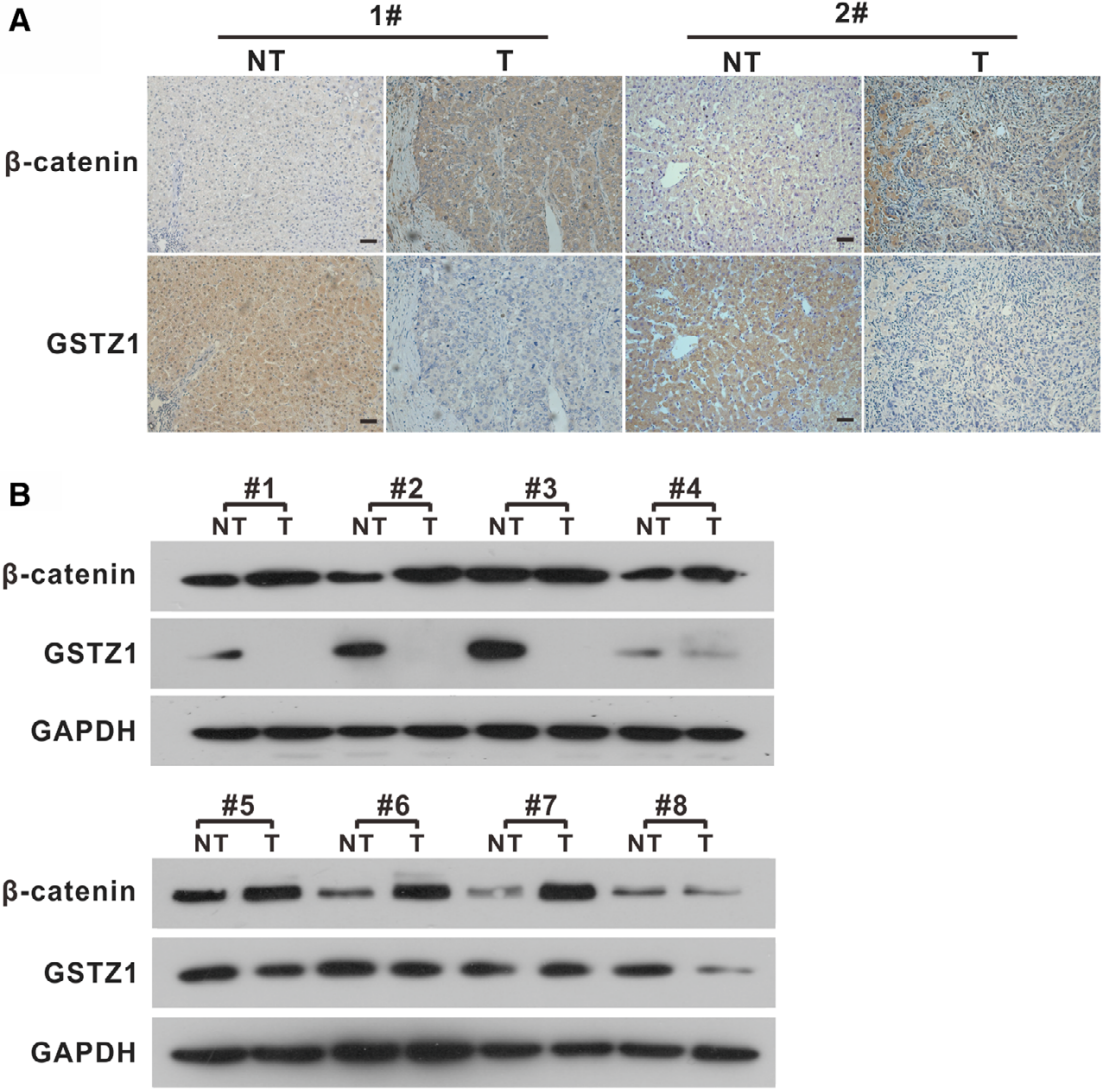
Validation of the negative correlation between GSTZ1‐1 expression and the Wnt/β‐catenin signalling pathway in HCC samples. (A) Representative images of IHC staining of β‐catenin and GSTZ1‐1 in paired HCC and NT. Scale bars, 50 μm. (B) Western blot analysis of β‐catenin and GSTZ1‐1 in paired HCC and paired NT samples. T, paired tumour tissues.

To further investigate the clinical relevance of GSTZ1‐1 and the Wnt/β‐catenin pathway, we performed IHC analyses to assess the co‐existence of GSTZ1‐1 and β‐catenin in paired tumour and nontumour liver tissues from patients. We found that β‐catenin was expressed at low levels in the NT but was significantly highly expressed in tumours, exhibiting expression patterns exactly opposite of those of GSTZ1‐1 (Fig. 7A). We further detected the protein expression levels of β‐catenin and GSTZ1‐1 and found they were negatively correlated in human HCC tissues (Fig. 7B). Together, our data suggest that GSTZ1‐1 may negatively regulate Wnt/β‐catenin pathway in HCC tissues.

## Discussion

Glutathione S‐transferases (GSTs) play various roles in xenobiotic and endogenous compound metabolism. Human genetic diseases have been reported that correspond to deficiencies in all enzymes in this pathway, with the exception of the penultimate enzyme, GSTZ1‐1 23. GSTZ1‐1, a member of the GSTs family that is widely distributed among many species, has essential functions in Phe metabolism. The previous study confirmed that GSTZ1‐1 deficiency is associated with poor prognosis in HCC.

In addition to the modulation of xenobiotic metabolism, GSTs are also tightly associated with the regulation of cellular signalling pathways. Class Mu and Pi GSTs have been reported to inhibit Ask1 and JNK by physically interacting with these kinases 24, 25. Moreover, GSTs deficiency has been reported to be involved in tumorigenesis. For example, *Gstp1/p2* ^−/−^ mice show approximately threefold more papillomas than controls 26. *Gstz1* ^−/−^ mice develop liver necrosis when administered 3% Phe in drinking water 27. These previous findings strongly suggested that GSTs may participate in tumorigenesis by regulating cellular pathways, but the exact molecular mechanisms remain unknown.

The RNA‐Seq data obtained in this study provide comprehensive expression profiles of GSTZ1‐1‐overexpressing Huh7 cells compared to control cells. The GO analysis results revealed the significant biological processes associated with the DEGs mainly included small molecule metabolic processes and xenobiotic metabolic processes. Our pathway interaction network analyses showed that most of the metabolic pathways were upregulated, while some oncogenic signalling pathways including the Wnt/β‐catenin pathway were downregulated. These results suggest that GSTZ1‐1 might suppress hepatocarcinogenesis and HCC progression by regulating metabolic programmes and downregulating relevant oncogenic signalling pathways.

Wnt/β‐catenin signalling is commonly aberrantly active in HCC 22. β‐Catenin, a core component of this signalling pathway, is bound to a multiprotein degradation complex comprising casein kinase I, glycogen synthase kinase 3β, adenomatous polyposis coli protein and axin 28. When Wnt proteins bind to cell‐surface receptors (frizzled) and co‐receptors, the canonical Wnt pathway is induced and β‐catenin accumulates in the cytoplasm and translocates to the nucleus 29, where it promotes the transcription of genes involved in cell proliferation, migration and metastasis 30, 31. Anneke *et al.* showed that GSTZ1‐1 deficiency leads to GSH depletion and oxidative stress 32. ROS may augment Wnt/β‐catenin signalling by mediating the redox‐dependent interaction between nucleoredoxin and dishevelled 33, 34. In our present work, we showed that GSTZ1‐1 can suppress β‐catenin expression and consequently Wnt/β‐catenin signalling. Therefore, we speculate that this regulation may be mediated by ROS, but the molecular mechanism remains to be further studied.

In summary, our transcriptomic results indicate, for the first time, that GSTZ1‐1 can downregulate Wnt/β‐catenin signalling in hepatoma cells. This study broadens our understanding of the biological function of GSTZ1‐1, which may be helpful in further elucidating the underlying molecular mechanism by which GSTZ1‐1 acts as a tumour suppressor in the context of HCC.

## Conflict of interest

The authors declare no conflict of interest.

## Author contributions

NT and KW conceived the study and modified the paper. CL completed the experiments, conducted the data analysis and drafted the manuscript. QJW helped with the data analysis.

## Data Availability

The gene expression data have been deposited in the Gene Expression Omnibus (GEO) database under accession number http://www.ncbi.nlm.nih.gov/geo/query/acc.cgi?acc=GSE117822.
